# Development of a Sustainable Bone Regeneration Material Using Apatite Paste Derived from Eggshell Waste

**DOI:** 10.3390/jfb16060201

**Published:** 2025-06-01

**Authors:** Masatsugu Hirota, Chihiro Mochizuki, Toshitsugu Sakurai, Hiroyuki Mishima, Chikahiro Ohkubo, Takatsugu Yamamoto

**Affiliations:** 1 Department of Education for Dental Medicine, Tsurumi University School of Dental Medicine, 2-1-3, Tsurumi, Tsurumi-ku, Yokohama 230-8501, Kanagawa, Japan; sakurai-toshitsugu@tsurumi-u.ac.jp (T.S.); yamamoto-tk@tsurumi-u.ac.jp (T.Y.); 2 Department of Dental Engineering, Tsurumi University School of Dental Medicine, 2-1-3, Tsurumi, Tsurumi-ku, Yokohama 230-8501, Kanagawa, Japan; c.mochizuki@bioapatite.jp (C.M.); mishima6065@circus.ocn.ne.jp (H.M.); 3 BIOAPATITE Inc., 1-5-3, Shiromachi, Hikone 522-0068, Shiga, Japan; 4 Department of Oral Rehabilitation and Prosthodontics, Tsurumi University School of Dental Medicine, 2-1-3, Tsurumi, Tsurumi-ku, Yokohama 230-8501, Kanagawa, Japan; okubo-c@tsurumi-u.ac.jp; 5 Department of Applied Biological Chemistry, Graduate School of Agricultural and Life Sciences, The University of Tokyo, 1-1-1, Yayoi, Bunkyo-ku, Tokyo 113-8657, Japan; 6 Department of Operative Dentistry, Tsurumi University School of Dental Medicine, 2-1-3, Tsurumi, Tsurumi-ku, Yokohama 230-8501, Kanagawa, Japan

**Keywords:** apatite, eggshell, bone formation, magnesium, μ-computed tomography (CT)

## Abstract

Apatite pastes derived from eggshell waste (BAp) were implanted onto the calvarial bone of rats, and bone formation was evaluated using X-ray μ-computed tomography (CT) and histological evaluation. BAp was mixed with distilled water to prepare a paste. Monoclinic hydroxyapatite of mineral resources (HAp) was used as a control. A 5 mm diameter PTFE (polytetrafluoroethylene) tube was filled with apatite pastes and implanted in the calvarial bone of 9-week-old Sprague Dawley rats for 8 weeks. A larger radiopaque area, similar to that of native bone, was observed in the BAp paste-implanted specimens than that of HAp paste. The bone mineral density (BMD) value of the BAp paste was significantly higher than that of the HAp paste (*p* < 0.05). In the histological evaluation, new bone formation was noticed from the calvarial side for both apatite specimens, and HAp remained in the PTFE unlike BAp. The bone mass (BM) value of the BAp paste was significantly higher than that of the HAp paste (*p* < 0.05). SEM and XRD analyses revealed that BAp was microcrystalline and poorly crystalline. The promotion of new bone formation may contribute to the crystallinity and Mg content of BAp. BAp was found to be useful as a bone regeneration material.

## 1. Introduction

Progress in material development technologies is ecologically and socially important, because developing new materials can contribute to sustainable development goals (SGDs) in various ways [1]. Utilizing waste resources is crucial in the medical and dental fields and plays a key role in the economic and sustainable development toward social wellbeing.

Hydroxyapatite is widely known as an excellent bone-substitute material, and most hydroxyapatite materials are chemically synthesized. By contrast, hydroxyapatite derived from natural sources, e.g., seashell waste, that has been recycled has also been applied in various fields, including biomaterials [2]. Vecchio et al. [3] reported that they obtained high-strength hydroxyapatite as a bone regeneration material by the hydrothermal reaction of powdered conch shell and giant clams as the calcium carbonate source. Zhang et al. [4] also reported that coral shell and cuttlebone were hydrothermally converted to HA. Fernández-Penas et al. [5] synthesized hydroxyapatite micro/nanoparticles from oyster shell waste using a low-temperature hydrothermal method and reported that their physicochemical characteristics and biological compatibility make them a useful osteoinductive platform for bone tissue engineering. Wu et al. [6] prepared hydroxyapatite powder using oyster shells and dicalcium phosphate dihydrate as raw materials via ball milling and heat treatment. In dentistry, dentine derivatives from the extracted teeth have been investigated as bone-regenerated material [7]. Dentin mainly contains hydroxyapatite as inorganic component (approximately 65%) and collagen as organic component (approximately 35%) [8]. Bianchi et al. [9] reported the effectiveness of dentin derivatives on cell behaviors.

The pure hydroxyapatite powder contained Mg and Sr as trace elements. However, the composition of apatite derived from shellfish varies, because the Ca concentration varies depending on the seawater environment in which the shellfish grow. Furthermore, there are concerns regarding the formation of sulfates and impurities during firing.

We focused on hydroxyapatite synthesized by regenerating Ca from discarded eggshells of edible chicken eggs. Japan consumes 2.5 million tons of eggs, generating 250,000 tons of eggshells, all of which is discarded as food waste. Because chickens consume controlled composition fertilizers, the apatite derived from their eggshells has a stable composition. With regard to applications for biomaterials, apatite derived from eggshells has been investigated as a bone regeneration material in several previous studies [10,11,12]. On the basis of the above-mentioned, we developed a new technology to synthesize natural hydroxyapatite from all discarded eggshells and convert them into a sustainable resource. Our apatite derived from eggshells synthesized using this new technology, which we call bioapatite, can be prepared without the use of buffers, dissolving agents, or catalysts. An original method and apparatus were used to separate the eggshell and eggshell membrane, and the eggshell was powdered into microcrystals [13]. Bioapatite has low toxicity and is not be harmful to the human body, and it has already been commercialized as an ingredient in toothpaste to remove stains because of its protein adsorption ability. In addition, the remineralization effect of bioapatite was confirmed by measuring the degree of mineralization [13].

Bioapatite has a higher inorganic element content than that of mineral resource apatite. Bioapatite was analyzed for trace element concentrations using inductively coupled plasma (ICP) emission spectroscopy. The ICP emission spectroscopy results for bioapatite revealed that the Mg content was 2 ppm for apatite mineral resources and 2133 ppm for bioapatite. Elements other than Mg, such as Na and K, were also present in higher concentrations [14]. Mg is an essential element found in the human body at relatively high concentrations and is known as an inorganic element with low toxicity. Furthermore, Mg ions have been reported to affect bone metabolism and promote bone formation [15,16,17,18].

Thus, the use of eggshell-derived apatite bone regeneration material may contribute to sustainability and improve bone compatibility. In this study, we aimed to develop a new bone regeneration material, and apatite pastes derived from eggshells were implanted onto the calvarial bone of rats. The bone augmentation effect was evaluated using X-ray μ-computed tomography (CT) and histological evaluation. It is expected that BAp will be applicable for dental clinics.

## 2. Materials and Methods

### 2.1. Materials and Apatite Synthesis

Sulfate-free apatite derived from eggshells (BAp; BioApatite-Medical, BIOAPATITE Inc., Hikone, Japan) was used as received. BAp is synthesized from eggshell waste as a calcium–containing raw material by an ion-exchange reaction. BAp was used as the apatite of natural origin for the experimental group, whereas monoclinic hydroxyapatite (HAp; FUJIFILM Wako Pure Chemical Corp., Osaka, Japan) was employed as the apatite from mineral resources for the control group.

Eggs obtained from domestic chickens were cracked and immediately separated into eggshells and eggshell membranes using an original apparatus (BIOAPATITE Inc.) [13]. First, calcium oxide was obtained by calcining calcium carbonate derived from eggshells in an electric furnace. Afterwards, calcium hydroxide was obtained from calcium oxide and then converted to hydroxyapatite following reaction. A total of 148 g of calcium hydroxide was mixed with 500 mL of pure water. Next, 1 L of 3 mol/L hydrochloric acid was added until the solution became clear. Further, 7 mL of 1 mol/L barium chloride was mixed and was stirred for 15 min. Suction filtration was performed on the white turbid solution to obtain the filtrate. Tris hydrochloric acid buffer was added until the pH was neutral. The pH was adjusted to 11 by adding 4 mol/L sodium hydroxide to the solution. While maintaining a pH of 9 or higher, dipotassium hydrogen phosphate (0.6 mol/L) was added dropwise to the resulting solution. After the required amount of dipotassium hydrogen phosphate was added dropwise, this solution was stirred for at least 2 h. The resulting liquid was subjected to suction filtration and washed out by applying a large quantity of water. Finally, obtained slurry was dried and ground in a jet mill (BIOAPATITE, Inc.).

### 2.2. Apatite Characterization

Scanning electron microscope (SEM; JCM6000, JEOL Ltd., Tokyo, Japan) was used to observe the apatite crystals of BAp. The BAp powder was placed directly onto a conductive tape. Au-sputter-coated BAp was observed at an accelerating voltage of 10 kV.

A laser diffraction particle size analyzer (SALD–2200, SHIMADZU Corp., Kyoto, Japan) was applied to analyze the particle size distribution of the BAp powder. From the particle size distribution, the average size of BAp was determined.

X-Ray diffraction (XRD, RINT2000, Rigaku Corp., Tokyo, Japan) was used to characterize the crystal structure and crystallinity of BAp. The X-ray source was Cu Kα which operated at 40 kV and 40 mA. Fourier transform infrared spectrophotometry (FTIR; ALPHA II, Bruker Corp., Billerica, MA, USA) was performed by the KBr method at a constant resolution of 2 cm^−1^ to analyze the composition of BAp.

Trace elements contained in BAp and HAp were analyzed using ICP-mass spectrometry (ICP-MS; ICPS-8100, SHIMADZU Corp., Kyoto, Japan). Magnesium, sodium (Na), and potassium (K) calibration standards of 1000 ppm were employed.

The amount of released Ca ions from BAp or HAp was measured using a compact Ca ion meter (LAQUAtwin-Ca-11, HORIBA Advanced Techno Co., Ltd., Kyoto, Japan). A total of 1000 mg of BAp or HAp powder was suspended in 50 mL of double-distilled water. Each suspension was stirred for 1 min and then ultrasonically treated. Afterwards, each suspension was stored in a thermostatic chamber at 37 °C. The Ca ion concentrations released in the supernatant were measured on days 1, 5, and 7 after immersion. Three run measurements were performed for BAp or HAp.

### 2.3. Animal Surgery

Animal surgery experiments were conducted in accordance with the Animal Experimental Ethical Guidelines of the Tsurumi University School of Dental Medicine (certificate nos. 22A006, 23A019, and 24A003).

A total of 8 male Sprague Dawley rats (Slc:SD, Japan SLC Inc., Shizuoka, Japan), each weighing approximately 270–370 g and 9 weeks old, were used. The rats were brought to the animal experiment facility of Tsurumi University at 8 weeks old to acclimate to the experimental location. Two rats were housed in one cage in a temperature-controlled room at 20–25 °C with a 12 h alternating light–dark cycle and were allowed to consume water and a stock diet (CE–2, Nippon Formula Feed Manufacturing Co., Ltd., Yokohama, Japan) ad libitum during the experiment. Every animal was administered with a single dose. A total of 8 pastes were implanted for an 8-week implantation period, i.e., four BAp and four HAp pastes (*n* = 4, Sample size was instructed by the Animal Experimental Ethical Guidelines of the Tsurumi University School of Dental Medicine).

**Figure 1:**
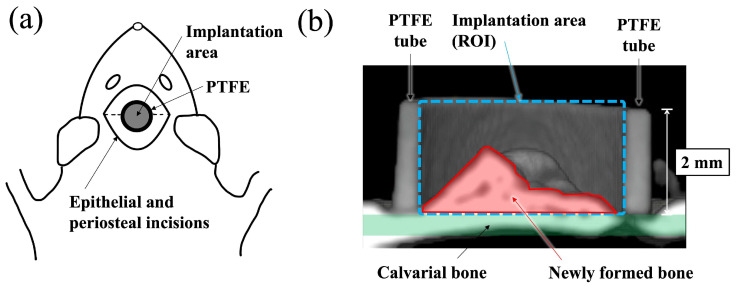
Schematic illustration for the implantation area and the region of interest (ROI). (**a**) Schematic illustration of apatite paste placement into the PTFE tube on rat calvaria from the top view. (**b**) Schematic diagram showing the ROI of a cross-sectional μ-CT view of a paste filled PTFE tube apatite paste implanted into periosteum pockets on of rat.

A schematic diagram of the surgical procedure used in this study is shown in Figure 1. Polytetrafluoroethylene (PTFE) tube containing apatite paste was placed into the space between the periosteum and the calvarial bone of the rat according to the previously reported method [19,20,21,22]. Before surgery, all apatite and PTFE tubes were sterilized with ethylene oxide gas. Surgery was performed under general anesthesia, which was induced by an intraperitoneal injection of 0.6 mg/kg ketamine hydrochloride (Daiichi Sankyo Propharma Co., Ltd., Tokyo, Japan). Then, 0.3 mg/kg medetomidine hydrochloride (Nippon Zenyaku Kogyo Co., Ltd., Fukushima, Japan) was used for sedation and analgesia, and the experimental animals were awakened using 0.15 mg/kg atipamezole hydrochloride (Nippon Zenyaku Kogyo Co., Ltd., Fukushima, Japan). A total of 150 mg of apatite powders was mixed with 160 μL of distilled water to form a paste (BAp paste or HAp paste). The paste was filled into a PTFE tube (inner and outer diameter: 3 mm and 4 mm, height: 2 mm) immediately after preparation. An incision was made at the top of head by a scalpel, and the periosteal flaps were elevated. Paste-filled PTFE tubes were put directly on the surface of calvarial bone. Decortication of the calvarial bone was not performed before insertion of the materials. After placing the PTFE tube into the periosteum pocket, the periosteal flap was returned and sutured over the surface of the PTFE tube using 4-0 nylon sutures. The rats were subcutaneously injected with 0.01 mg/kg latamoxef sodium (Shiomalin; SHIONOGI Co., Ltd., Osaka, Japan) to prevent infection after surgery.

### 2.4. Evaluation of Bone Formation

The paste-filled PTFE tubes and the surrounding bone tissue were immediately excised 8 weeks after implantation. After removal of the excess tissue, harvested specimens were fixed in 10% formalin neutral buffer solution (pH 7.4) for 7 days.

New bone formation was evaluated using μ-CT and histological evaluation. First, new bone formation into the PTFE was observed using a micro focus X-ray system (inspeXio SMX–225CT, SHIMADZU Corp., Kyoto, Japan) at 140 kV × 70 μA. The imaging conditions were 1024 × 1024 pixels, 1200 views, slice thickness 0.077 mm, and interslice distance 0.047 mm. Software (TRI/3D–BON–FCS64, ver. R.10.01.00.1-H-64, RATOC System Engineering Co., Ltd., Tokyo, Japan) was used to construct and analyze three-dimensional (3D) images of the obtained tomographic images. After μ-CT observation, the bone mineral density (BMD) value in the ROI (region of interest) inside the PTFE tube, as shown in Figure 1, was measured using CT values. As previously reported, ranges with a brightness similar to the CT value of the calvarial bone were defined as new bone [19].

After μ-CT observation, the specimens were dehydrated using ethanol (70%, 80%, 90%, 96%, and 100% in succession) and embedded in methyl methacrylate. Non-decalcified sections were prepared with approximately 60–80 μm thickness using a cutting–grinding machine (EXAKT–Cutting Grinding System, BS–300CP band system and 400 CS microgrinding system, EXAKT Co., Norderstedt, Germany) [23]. From each apatite specimen, one to two sections were obtained at the center of the implant materials. The sections were then stained with methylene blue and basic fuchsin and histologically evaluated using a light microscope (Eclipse Ni, Nikon Co., Ltd., Tokyo, Japan). Descriptive evaluation and histomorphometric analyses were performed. Newly formed bone area in the PTFE tube was determined as the bone mass (BM) value using an image analysis system (WinROOF, Visual System Division, Mitani Corp., Tokyo, Japan). The BM values were defined as the percentage of new bone range within the ROI, as shown in Figure 1b.

### 2.5. Statistical Measurement

Statistical significance was determined using SPSS for Windows (SPSS Statics 17.0; IBM Corp., Chicago, IL, USA). The 5% significance level was set. A non-paired *t*-test was employed to compare the data obtained from the amount of released Ca ion, BMD and BM values from histomorphometric measurements. The results are described as the mean value standard deviation (SD).

## 3. Results

### 3.1. Apatite Characterizations

**Figure 2:**
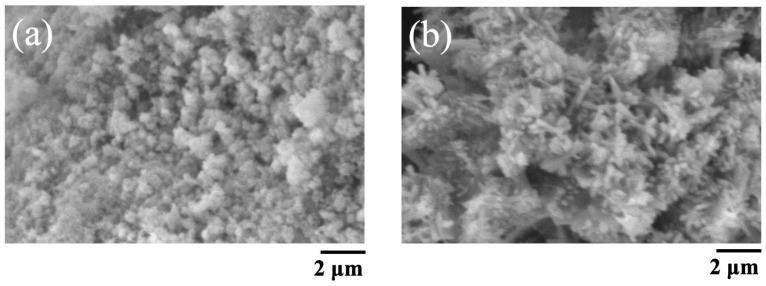
SEM pictures of BAp and HAp. (**a**) BAp, (**b**) HAp.

The SEM images of BAp and HAp are shown in Figure 2. The BAp crystals were finer than those of HAp. HAp has needle-like crystals, whereas BAp has spherical microcrystals.

**Figure 3:**
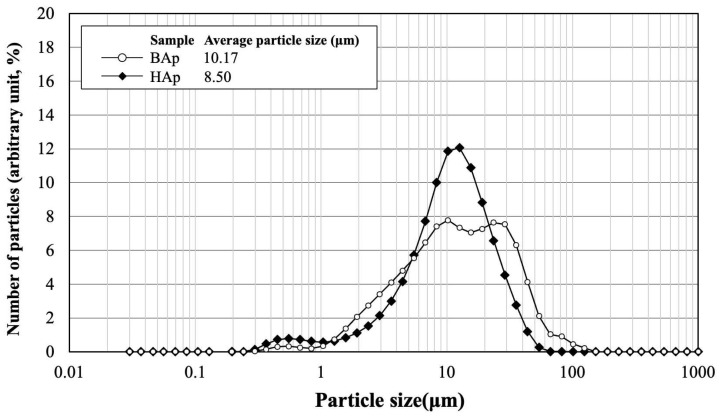
Particle size distribution and average size of BAp and HAp.

The particle size distribution and average size of BAp and HAp are shown in Figure 3. The average particle size was 10.17 μm for BAp and 8.50 μm for HAp. HAp exhibited a slightly lower value than that of BAp. However, particle size distribution was wider for BAp than for HAp. This indicates that there was a greater variation in the particle size of BAp.

**Figure 4:**
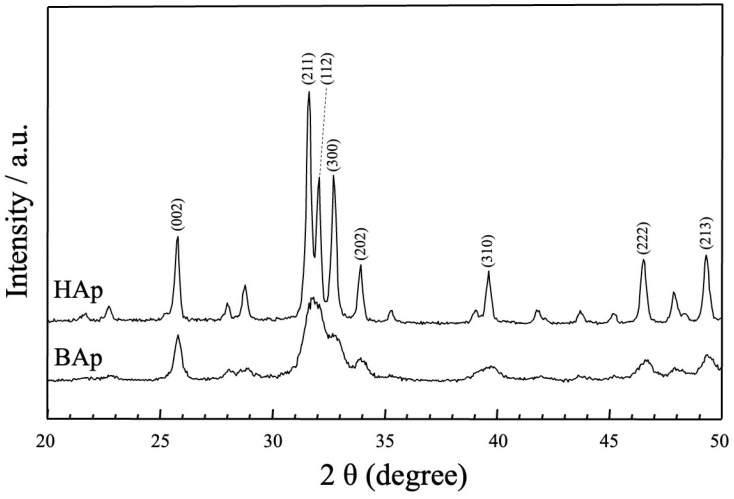
XRD patterns of apatite derived from BAp and HAp.

The XRD patterns of the apatite derived from BAp and HAp are shown in Figure 4. The phase compositions of BAp and HAp were characterized using X-ray diffraction. For HAp, sharp peaks of apatite could be observed at approximately 25.80°, 31.76°, 32.26°, 32.83°, 34.00°, 39.80°, 46.70°, and 49.50° for 2θ corresponding to (002), (211), (112), (300), (202), (310), (222), and (213) planes of hydroxyapatite. In BAp, broad apatite peaks were observed at the same positions as those in the diffraction peaks of HAp, at approximately 2θ = 26 and 32–33°. Thus, BAp was identified as less crystalline than HAp owing to its lower peak intensity.

**Figure 5:**
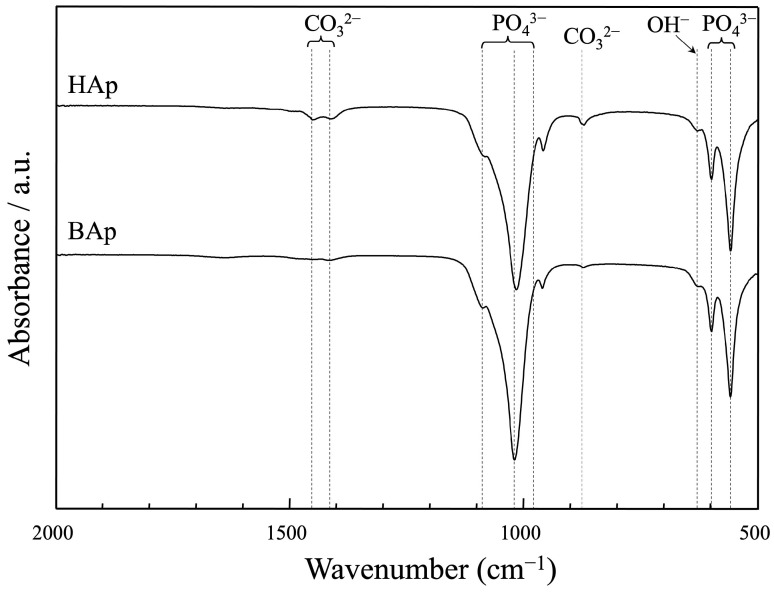
FT-IR spectra of apatite derived from BAp and HAp.

The FTIR spectra of apatite derived from BAp and HAp are shown in Figure 5. The bands at 633 cm^−1^ are characteristic of the hydroxyl group in the hydroxyapatite. The peaks at approximately 571, 602 cm^−1^, and 956, 1042, and 1096 cm^−1^ were assigned as phosphate groups of apatite. The peaks derived from carbonate ions were recognized at approximately 880, 1426, and 1457 cm^−1^ for both BAp and HAp. The carbonate content was lower in BAp than that in HAp because of the weaker peak intensity of the carbonate groups.

The trace element concentrations in BAp and HAp from analysis using an ICP-MS is shown in Table 1. Elements Mg and Na were detected in BAp at concentrations of nearly 2000 ppm. Mg and Na were almost absent in HAp.

**Figure 6:**
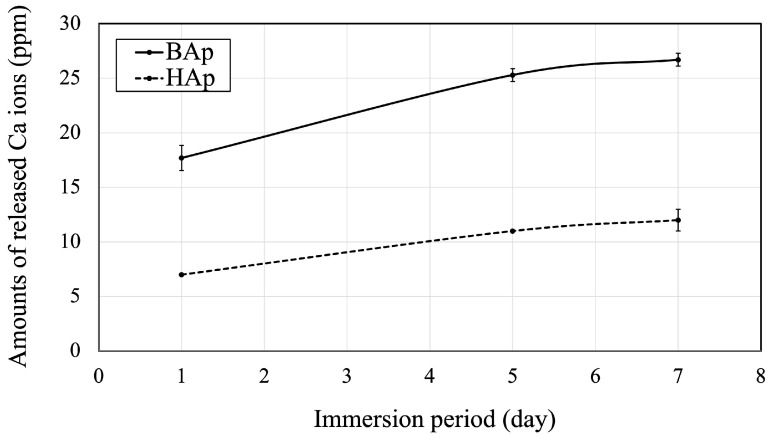
Released Ca ions from BAp and HAp.

The amounts of released Ca ions from BAp or HAp are shown in Figure 6. The amount of Ca released from BAp after 1, 5, and 7 days of immersion was significantly higher than that from HAp (*p* < 0.05).

### 3.2. Micro-CT Evaluation

**Figure 7:**
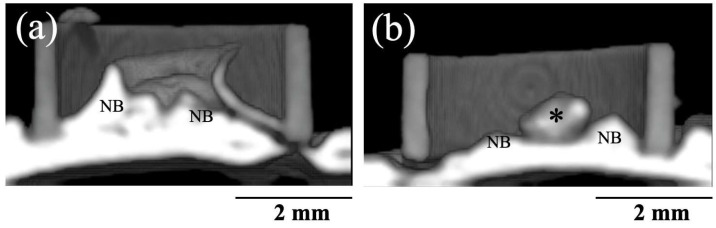
Three-dimensional CT images of new bone formations into PTFE tubes by implantation of BAp and HAp paste 8 weeks after surgery. (**a**) BAp paste, (**b**) HAp paste. NB: Newly formed bone, Asterisk: residual apatite.

Figure 7 shows 3D CT images of new bone formation in PTFE tubes by the implantation of BAp and HAp paste, 8 weeks after surgery. In both groups, new bone formation was observed on the calvarial side rather than the periosteal side. A larger radiopaque area similar to that of the original bone was observed on the calvarial bone in the BAp-implanted specimens than that in HAp-implanted specimens. Residual apatite was observed without absorption. The identity of residual apatite was confirmed because of its different permeability to that of the surrounding bone. More residual apatite was observed in HAp than that in BAp. Table 2 shows the percentages of BMD measured using the micro-CT images. In 8 weeks post surgery, the BMD of the BAp paste was significantly higher than the value of the HAp paste (*p* < 0.05).

### 3.3. Histological Observation

All the rats stayed healthy during the animal experimental period. There were no clinical inflammation signs or adverse tissue reactions in the surrounding tissues of any of the rat specimens, and all implants remained in situ.

**Figure 8:**
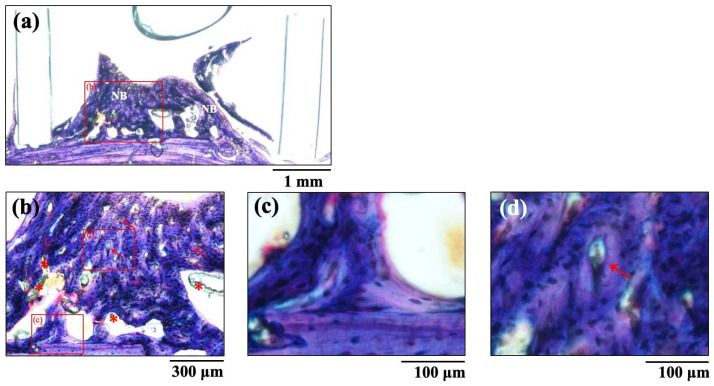
The histological images of new bone formation in the PTFE tubes by implantation of BAp paste 8 weeks after surgery. (**b**) Higher magnification images of the boxed area in (**a**). (**c**,**d**) Further higher-magnification images of the boxed areas of (**b**), respectively. NB: Newly formed bone, Arrow: Haversian canal, Asterisk: residual apatite.

**Figure 9:**
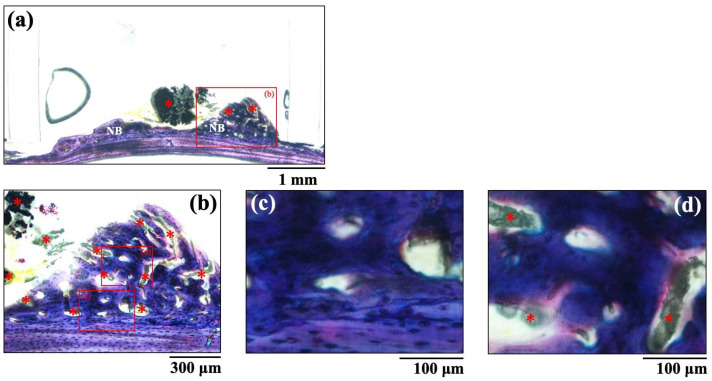
The histological images of new bone formation in the PTFE tubes by implantation of HAp paste 8 weeks after surgery. (**b**) Higher magnification images of the boxed area in (**a**). (**c**,**d**) Further higher-magnification images of the boxed areas of (**b**), respectively. NB: Newly formed bone, Asterisk: residual apatite.

The histological images of new bone formation in the PTFE tubes formed by the implantation of the BAp and HAp pastes, 8 weeks after surgery, are shown in Figure 8 and Figure 9. Histological appearances were consistent with those of the μ-CT observations. Newly formed bone was recognized on the calvarial side but not on the periosteal side in both BAp and HAp. Subperiosteal tissue implanted with the BAp paste displayed more new bone than that implanted with the HAp paste. More residual apatite (asterisks) was clearly recognized in the HAp-implanted specimens than in BAp-implanted specimens. All specimens displayed large numbers of osteocytes, sparse laminae, and irregular bone tissue with collagen fibers running in various directions. In other words, immature bone was observed in both BAp and HAp paste implantation groups. However, the presence of an external circumferential lamella was identified, as shown in Figure 8c, and the formation of Haversian canals was observed, as shown in Figure 8b,d (arrowhead), in the BAp paste-implanted specimens. By contrast, there was no formation of external circumferential lamellae, Haversian canals, or osteocytes, as shown in Figure 9c, in the HAp paste-implanted specimens. This indicates that more mature bone tissue was regenerated by calcification in the BAp paste-implanted specimens than that in HAp paste-implanted specimens.

The BM assessed histologically 8 weeks after implantation is shown in Table 2. These results are similar to those of the BMD results from the μ-CT images. The BM of the BAp paste was significantly higher than the value of the HAp paste 8 weeks after implantation (*p* < 0.05).

## 4. Discussion

In this study, apatite derived from eggshells was implanted as a bone regeneration material in a PTFE tube on the calvarial bone of rats. Bone formation was evaluated radiologically using μ-CT and histopathological examination of new bone formed on the lateral side after 8 weeks was performed. The mineral resource apatite was used as a control in an experimental design to evaluate the effect of apatite derived from eggshells on bone augmentation.

As described above, we focused on bioapatite, which contains inorganic elements that promote bone formation. As the first step of this study, a sulfate-free bioapatite was synthesized as a biomaterial, BAp, which is less likely to cause inflammation. In the animal experiments, no inflammatory reactions were observed in any rat. Regarding allergies of apatite derived from eggshell, Ebisawa et al. [24]. reported that non-calcinated eggshell Ca contains almost no contamination of egg white, and that the allergenic activity of non-calcinated eggshell Ca is similar to those of calcinated eggshell Ca. Bioapatite can be useful in industrial applications and in the field of biomaterials. Moreover, bioapatite has high industrial productivity and can be supplied to the market at a low cost because of the stable supply of raw materials in Japan. Therefore, it meets the concept of SGDs and is expected to be applied to a variety of materials for next-generation artificial apatites.

External bone augmentation using a subperiosteal pocket model of rat calvaria was applied to evaluate new bone formation in this study. Previous experiments using this model [19,20,21,22] suggested that the initiation site of bone formation is on the calvarial surface and not the periosteum, which is the same as that in the present study. The periosteum supposedly stimulates vascularization, deregulates osteoblast differentiation, and promotes new bone formation.

The μ-CT images and histological appearances revealed that immature bone tissue was formed in both BAp and HAp. Histological images of the new bone revealed that BAp exhibited greater bone formation and a higher degree of mineralization than that of HAp. These histological findings are consistent with the statistical evaluation of BMD and BM values. Although most HAp remained, most BAp was absorbed and replaced by new bone formed from the host bone. This finding suggests that BAp is a bioresorbable osteoconductive material.

SEM revealed that the BAp crystals were finer than the HAp crystals. Nano-hydroxyapatite has recently received considerable attention in biomaterial research because of specific characteristics such its superior bioabsorbability, osteoconduction, and osteoinduction compared with that of conventional macro- and micro-sized hydroxyapatite [25,26]. The crystal size of BAp used in this study may affect bone formation. Furthermore, XRD measurements showed that BAp had a wide peak intensity than that of HAp, indicating low crystallinity. A greater amount of released Ca ion from BAp was due to the smaller particle size and low crystallinity. Hayakawa et al. [27] synthesized hydroxyapatite with different crystallinities from Ca-EDTA (ethylenediaminetetraacetic acid) complexes, composited them with the biodegradable polymer PLGA (Polylactic-co-glycolic acid), and evaluated biocompatibility. PLGA/apatite porous composites made of low-crystalline apatite progressed new bone formation in the tibia cortical defects of rabbit compared with a composite made of high-crystalline apatite. Thus, the low crystallinity of BAp may contribute to the promotion of new bone formation.

BAp contains more inorganic elements than HAp, especially a larger amount of Mg as shown in Table 1. Mg in the biomaterial has promoted new bone formation in the surrounding tissue [15,16,17]. In vivo and in vitro reports have concluded the osteogenic ability of Mg alloys [18]. Odashima et al. [28] performed an 8-week implantation in rat tibia to investigate the osteoconductive effects of Mg. The researchers reported that SEM/EDX (energy dispersive X-ray spectroscopy) analyses confirmed the increase in the concentrations of Ca and P on the Mg surface, and the tissue specimen showed newly formed bone on the surface of near the implanted bone. In this study, quantitative analysis using thermogravimetry–differential thermal analysis (TG-DTA) was necessary for matters related to carbonate groups. Quantification of the eluted ions and immunohistological evaluations are required. Recently, carbonate apatite has been identified as a bone-regeneration material that is rapidly absorbed and replaced by bone. Ishikawa et al. [29] and Mano et al. [30] fabricated bone regeneration materials of carbonate apatite by immersing a calcium carbonate block and evaluated bone formation by grafting carbonate apatite granules into bone defects adjacent to dental implants inserted into the jawbone of beagle dogs. In this study, the peak intensity in the BAp FTIR spectra was weaker than that of HAp. This suggested that the carbonate content of BAp was low. Some elements of eggshell apatite, other than the carbonate content, may have affected the bone-remodeling cycle. Nevertheless, the mechanisms involved in BAp formation should be investigated in more detail.

In this study, the specimens were provided as a paste mixed with water, which could be used for any bone defect by adjusting the consistency. The viscosity of the apatite paste can be obtained using water without any reagents and provided on the dental chair side immediately before treatment. The present apatite paste technique has the potential to provide easy and widespread applications in dental clinics. The use of a simple paste allowed for a facile evaluation of the bone formation potential [19]. This clearly indicates that BAp had higher new bone formation capability than that of HAp in this study. However, composites of apatite with other materials and their shapes are expected to be developed. The clinical application of BAp as a bone regeneration material faces challenges, such as its behavior as a biomaterial and biological response, and this study serves as fundamental evidence.

Finally, BAp from eggshells, which are waste resources, has environmental and economic benefits and is a promising material as bone-regenerating material in dental clinics. However, this study still has limitations for dental clinical application. In addition to quantitative analysis, it is necessary to measure the amounts of other ions released, such as Mg ions. To understand the mechanism, in vitro evaluation requires cell culture assays to evaluate osteoblast activity and cytocompatibility. In animal studies, longer-term implantation using larger sample sizes studies are needed.

## 5. Conclusions

The results obtained in this study showed that apatite derived from eggshells may be absorbed and replaced by bones. BAp, obtained from discarded eggshells, showed low crystallinity and microcrystallinity. Compared to mineral resources, hydroxyapatite and μ-CT displayed more new bone, similar to native bone, and higher BM values were obtained on histological observation. The crystallinity and Mg content of BAp may have contributed to the promotion of bone formation. Future work will include studies on practical applications such as composites and absorbency control. Therefore, it can be suggested that that apatite derived from eggshells may be useful as a bone regeneration material.

## Figures and Tables

**Table 1:** The trace element concentrations in BAp and HAp from analysis using an ICP-MS (ppm).

Elements	Mg	Na	K
BAp	1974	1997	41
HAp	2	0	7

**Table 2:** Percentage of bone mineral density (BMD) measured by μ-CT and new bone mass (BM) values were assessed histologically 8 weeks after implantation.

Specimen (Bone Filling Material)	BAp Paste	HAp Paste
BMD (%)	32.53 (6.57) ^a^	20.95 (1.37) ^a^
BM (%)	24.82 (6.42) ^b^	16.02 (3.04) ^b^

Values in parentheses are SD. The same superscripts indicate significant differences (*p* < 0.05).

## Data Availability

The original contributions presented in the study are included in the article, further inquiries can be directed to the corresponding author.

## Abbreviations

The following abbreviations are used in this manuscript:

SDGs	Sustainable development goals
ICP	Inductively coupled plasma
CT	Computed tomography
BAp	Sulfate-free apatite derived from eggshells waste for the experiment group (BioApatite-Medical)
HAp	Monoclinic hydroxyapatite as the apatite from mineral resources for the control group
SEM	Scanning electron microscope
XRD	X-Ray diffraction
FTIR	Fourier transform infrared spectrophotometry
PTFE	Polytetrafluoroethylene
BMD	Bone mineral density
BM	Newly formed bone mass
ROI	Region of interest
SD	Standard deviation
EDTA	Ethylenediaminetetraacetic acid
PLGA	Polylactic-co-glycolic acid
SBF	Simulated body fluid
EDX	Energy dispersive X-ray spectroscopy
TG-DTA	Thermogravimetry-differential thermal analysis
